# Prevalence of infertility in Sudan: A systematic review and meta-analysis

**DOI:** 10.5339/qmj.2021.47

**Published:** 2021-10-01

**Authors:** Abdullah A. A., Musa Ahmed, Adesina Oladokun

**Affiliations:** ^1^Department of Reproductive Health Sciences, Pan African University Life and Earth Sciences Institute (PAULESI), University of Ibadan, Ibadan, Nigeria E-mail: Bahlol32029@gmail.com; ^2^Department of Biomedical Sciences, University of Gadarif, Gadarif, Sudan; ^3^Department of Veterinary Surgery, Faculty of Veterinary Medicine, Al-Salam University, West Kordofan, Sudan; ^4^Department of Obstetrics and Gynecology, College of Medicine, University of Ibadan, Ibadan, Nigeria

**Keywords:** meta-analysis, primary infertility, prevalence, secondary infertility, Sudan, unexplained infertility

## Abstract

Background/aim: Infertility is defined as the inability of heterosexual couples to achieve a successful clinically recognizable pregnancy after 12 months or more of regular, unprotected sexual intercourse. Infertility estimations are very important to inform the healthcare policymakers and governments to implement appropriate social and economic policies. Thus, this study aimed to estimate the pooled prevalence of infertility (primary and secondary) and its etiologic factors in Sudan.

Methods: This study included all published and unpublished studies written in Arabic or English. Electronic sources (namely, PubMed, MEDLINE, Embase, Cochrane Central Register of Controlled Trials, and ClinicalTrials.gov) and nonelectronic sources (direct Google search, Google Scholar, OpenGrey, OATD, WorldCat log, and university websites) were used from their inception to May 16, 2021. A total of 1955 studies were reviewed, of which only 20 studies were eligible for the meta-analysis. Studies were eligible if they provided the prevalence of infertility in Sudan. The Joanna Briggs Institute Quality Assessment Tool was used to evaluate each study. Data synthesis and statistical analysis were conducted using Jeffrey's Amazing Statistics Program version 0.14.1.0.

Results: The pooled prevalence of overall infertility, primary infertility, and secondary infertility in Sudan were 13% (I^2^ = 96.45, *p* < 0.001), 65% (I^2^ = 98.5, *p* < 0.001), and 35% (I^2^ = 98.5, *p* < 0.001), respectively, and the prevalence of infertility factors were 41%, 27%, 16%, and 17% for female, male, combined factors, and unexplained factors, respectively. Women with infertility were mainly present because of ovulatory disorders (ovulatory factors, 36%; polycystic ovary syndrome, 38%). By contrast, spermatic disorders such as azoospermia (37%), oligozoospermia (30%), and asthenozoospermia (30%) were the main causes of male infertility.

Conclusion: In Sudan, the prevalence of primary infertility is higher than that of secondary infertility. Female factors were the most common causes of infertility in Sudan, and this study found a high prevalence of unexplained factors. Polycystic ovary syndrome and azoospermia were the most common causes of female and male infertility in Sudan, respectively. The interpretation of these findings should take into consideration the presence of substantial heterogeneity between the included studies.

## Introduction

Infertility is defined as the inability of heterosexual couples to achieve a successful clinically recognizable pregnancy after 12 months or more of regular, unprotected sexual intercourse.^1^ Primary infertility occurs when the couple fails to achieve pregnancy after at least 1 year of having unprotected sex, and secondary infertility occurs when they were able to get pregnant at least once before but were unable subsequently.^2^ The heterosexual couple may suffer from infertility because of one or more of the following factors: male factors that are mainly related to spermatic disorders, e.g., azoospermia, oligozoospermia, asthenozoospermia, and/or female factors such as ovulation disorders, tubal factors, uterine factors, and unexplained factors.^2–4^ However, the complexity of these factors is attributed to the variability in the prevalence and incidence of infertility globally.^3–6^ Worldwide, the average infertility prevalence is 10%.^1^ Evidences, collected from 195 countries and territories worldwide between 1990 and 2017, showed that infertility cases were increasing and the risk of becoming infertile is higher in women than in men. The age-standardized prevalence of infertility increased by 0.370% annually for women and 0.291% annually for men.^7^

The prevalence of infertility in Africa is higher than that in other continents and range from 20% to 35%.^5,8,9^ In addition, the pooled prevalence of primary and secondary infertility is 49.9% and 49.8%, respectively, and the main causes of infertility are oligospermia and pelvic inflammatory disease for men and women respectively.^9^

In Sudan, nowadays, because of difficult financial and socioeconomic circumstances, men and women are usually getting married at a relatively old age; therefore, infertility and subfertility are becoming serious issues.^10^ Studies of the infertility prevalence in Sudan have shown varying results, with significant difference in some cases.^4,11–29^ Evidences collected from Sudan showed that the prevalence of overall infertility widely varied from 10.4% to 18%,^16–18,22,29^ in addition to conflicting results about the distributions of infertility types in Sudan, where some studies have reported that primary infertility was the most dominant type.^4,11–15,18–22^ By contrast, secondary infertility was found to be more dominant in other studies;^16,17^ thus, there is a need to extensively examine and critically appraise the available literature to make a pool estimation of infertility in Sudan and to use this evidence to address the issue of infertility, make a proper healthcare plan, and design extension programs and treatment modalities. Thus, this study aimed to estimate the pooled prevalence of infertility (primary and secondary) and its etiologic factors in Sudan.

## Methods

### Eligibility criteria

All studies that (1) defined human infertility based on the standard definition of the World Health Organization (WHO), (2) reported the prevalence of infertility (primary/secondary) and related factors in Sudan, and (3) are published in Arabic or English were eligible for this study. Moreover, studies were not eligible (1) if they were reviews, editorials, letters, and animal studies and (2) if the full text was not available (even if the author/s were contacted by email, no feedback was received after 2 weeks).

### Information sources

This study was conducted according to the guidelines of Preferred Reporting Items for Systematic Reviews and Meta-Analyses (PRISMA). Relevant information has been retrieved from electronic and nonelectronic sources. Electronic sources, namely, PubMed, MEDLINE, Embase, Cochrane Central Register of Controlled Trials, and ClinicalTrials.gov, were used to retrieve published articles. Nonelectronic sources included direct Google search, Google Scholar, OpenGrey, OATD, WorldCat log, and university websites. All these databases were searched from their inception to May 16, 2021, for human studies published in English and Arabic.

### Search strategy

Boolean search terms (i.e., AND, OR, NOT) were used to develop the research strategy. The final search strategy included the use of titles/abstracts related to (((Infertility) OR (sterility) OR (subfertility) OR (childlessness)) AND ((prevalence) OR (epidemiology) OR (frequency)) AND (Sudan)) taken from the study questions. Nonelectronic sources were a combination of direct Google search, Google Scholar, OpenGrey, OATD, WorldCat log, and university websites. In addition, a manual search by the investigators was performed for the gray literature and unpublished thesis/papers.

### Selection process

**Figure 1.**
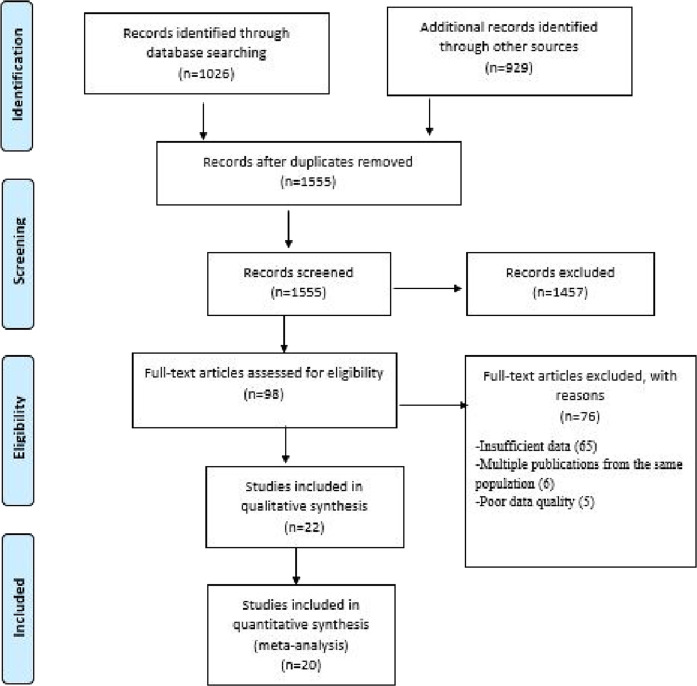
PRISMA flow diagram

First, all retrieved studies were exported to Mendeley citation manager version19.8 to check for duplication; duplicated articles were excluded from the study. Three authors (AA, MA, and SO) screened and evaluated the remaining studies independently by carefully reading their titles and abstracts. Each study that mentioned the outcomes of the review (infertility prevalence)/Sudan) in their titles and abstracts were considered for further evaluation based on the objectives, methods, participants, and key findings. Two authors (AA and MA) independently evaluated the quality of the relevant studies against the checklist. Any discrepancy was resolved by a discussion between the two authors (AA and MA) or by asking a third reviewer if consensus could not be reached. The selection process of the studies is presented using the PRISMA statement flow diagram (Figure 1).

### Data collection process

After the selection process of all appropriate articles for this systematic review and meta-analysis, relevant data were extracted by two investigators independently (AA and MA) using a data extraction template and presented through Microsoft Word 2016.

The investigators contacted the authors of any studies that do not report the aforementioned data (via email) to obtain the original data from them, and sensitivity analysis was performed for the data if the missing data were still not available after 2 weeks. The extraction template contained the authors’ name, publication year, study location, study design, sample size, infertility rate, prevalence of primary infertility, prevalence of secondary infertility, female factors, male factors, complained factors, and unexplained factors (Table 1). The data extraction accuracy was verified by comparing the data extraction results of the two investigators (AA and MA), who independently extracted the data in a randomly selected subset of papers (30% of the total).

### Data items

The main outcome of this study was the prevalence of infertility in Sudan, and it is measured by the direct report of the individual studies included in the analysis. To quantify the outcome, the investigators considered studies that reported the prevalence of infertility and the types of infertility in their statistics. From the total population, the result was interpreted by the proportions of the population that have any types of infertility.

### Study risk of bias assessment

Inclusion criteria were appraised for all retrieved articles using their titles and abstracts first, and the full text was screened to check the quality of each study before the final selection. The quality assessment criteria for the studies included in the current review were as follows: (1) The diagnosis of the infertile cases was based on WHO standards. (2) The couple had an infertility test. (3) Unexplained infertility was diagnosed after performing all available standard fertility tests. (4) The sample was representative of the population.

A comprehensive search (electronic/database search, manual search, gray literature search, and unpublished studies search) was performed to minimize the risk of bias. The risk of bias from the included studies was individually appraised by three investigators (AA, MA, and SO) using a critical appraisal tool (Joanna Briggs Institute Quality Assessment Tool).^30^ The publication bias for the included studies was checked by both visual inspection of the funnel plot and statistical symmetry of the funnel plot using Egger's regression test.

### Effect measures

In light of the study objectives, the proportion of infertility was used to synthesize and present the results for the analysis.

### Synthesis methods

Jeffrey's Amazing Statistics Program version 0.14.1.0 was used to synthesize and analyze the meta-analysis data. The recommendations of the I^2^ statistic described by Higgins et al.^31^ (with I^2^ of 75/100% and above suggesting considerable heterogeneity) were used to perform this meta-analysis. The result of this study (pooled proportion of infertility) was calculated using both the effect size, with a 95% confidence interval (CI), and standard error (SE). The effect size of this study was the prevalence of infertility (proportion), and it was calculated using binomial distribution. The SE was calculated using the sample size (n) and proportion of infertility (p) and was applied in the SE formula:  =  sqrt [p (1 − p)/n].

Potential publication bias was checked using a funnel plot and Egger's regression test, and the results were significant if the *p* values were  < 0.10. Sensitivity analysis and subgroup analysis were performed to determine the potential source of heterogeneity and possible source of bias. Studies were excluded from the final review if (1) they had a missing data and/or (2) they had a high risk of bias. The results were reported according to the PRISMA guidelines, and the findings of the included studies were first presented using a narrative synthesis, followed by a meta-analysis chart (Figure 1).

## Results

### Study selection

A total of 1955 articles were identified following search of major electronic, nonelectronic, and other relevant sources. A total of 400 articles were removed because of duplication, while 1555 studies were kept for further critical screening. From these studies, 1457 were excluded following a very careful screening of their titles and abstracts. From the remaining 98 articles, 76 were excluded because of inconsistency with the study inclusion criteria. Finally, 20 studies that fulfilled the eligibility criteria, involving 22379 participants, were included for the systematic review and meta-analysis. Figure 1 shows the selection process of the studies selected for the meta-analysis.

### Study characteristics

Of all included studies, 18 were cross-sectional studies, one was a cohort study, and the other was a descriptive prospective study. In addition, 13 studies were peer-reviewed studies, and the remaining seven were university theses. The prevalence of overall infertility in the Sudanese population was reported in only five studies, with a total of 18477 participants; meanwhile, the prevalence of primary and secondary infertility was examined by 18 studies with a pooled sample size of 5930 participants. Nearly half of the included studies were conducted in Khartoum state, and only three were on the national level. Table 1 shows the detailed characteristics of all included studies.

### Results of syntheses

**Figure 2.**
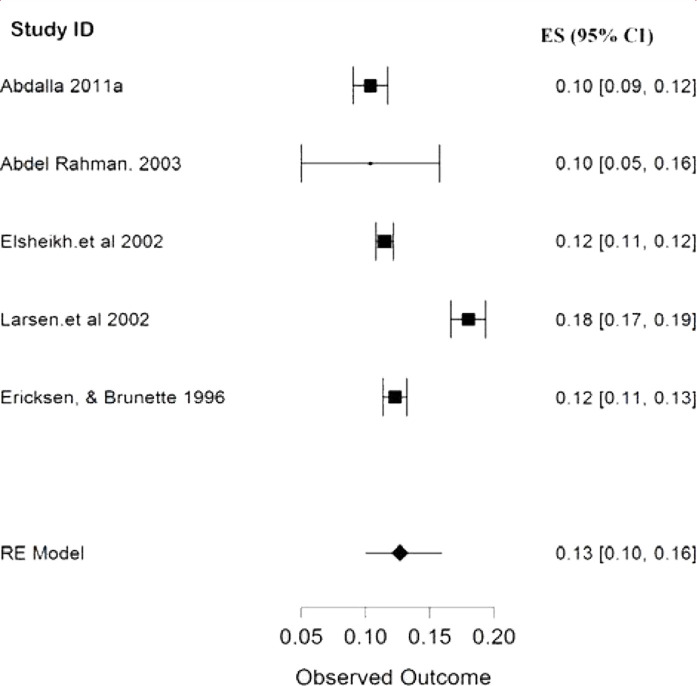
Forest plot (random-effects model) for the pooled prevalence of infertility in Sudan

**Figure 3.**
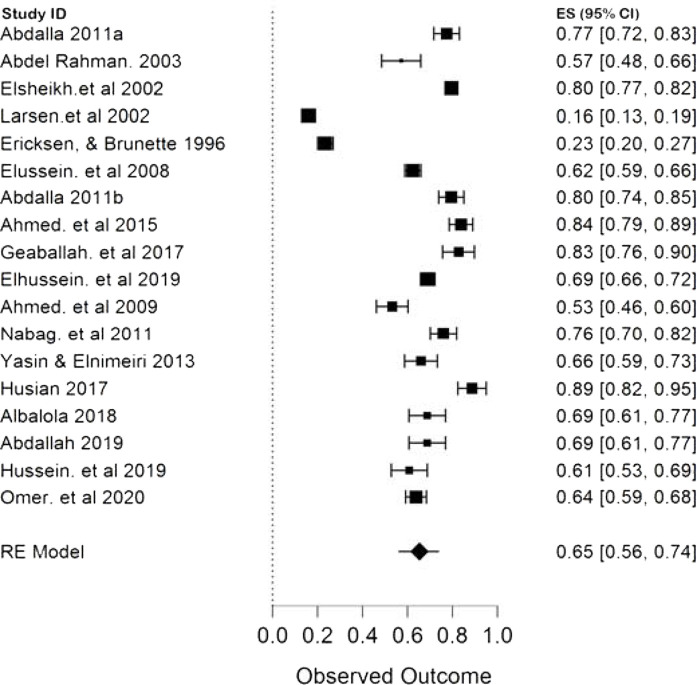
Forest plot (random-effects model) for the pooled proportion of primary infertility in Sudan

**Figure 4.**
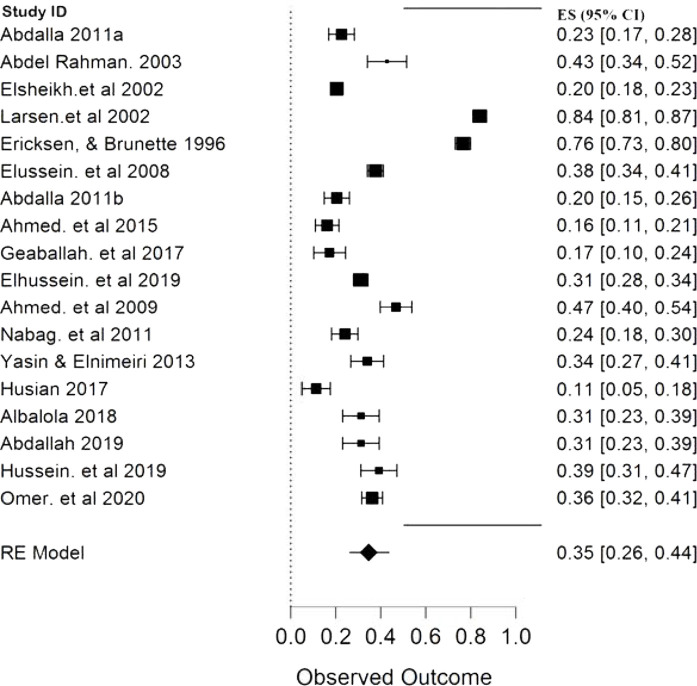
Forest plot (random-effects model) for the pooled proportion of secondary infertility in Sudan

The result of this meta-analysis showed that only five studies, with a pooled sample size of 18477 participants, provided the overall prevalence of infertility in Sudan, and between them, the heterogeneity was high with (*p* < 0.00001, I^2^ 96.45%). Therefore, the random-effects model was employed for the analysis. Finally, the pooled proportion of infertility in Sudan was 13% with a CI of 10%–16% (Figure 2). The pooled prevalence of primary and secondary infertility in Sudan from the remaining 18 studies were 65% (CI 56%–74%) (Figure 3) and 26% (CI 26%–44%) (Figure 4), respectively. For this analysis, the random-effects model was applied because the heterogeneity was substantially high (*p* < 0.00001, I^2^ 98.5%) for both primary and secondary infertility cases.

### Subgroup analysis of primary and secondary infertility by publication year

**Figure 5.**
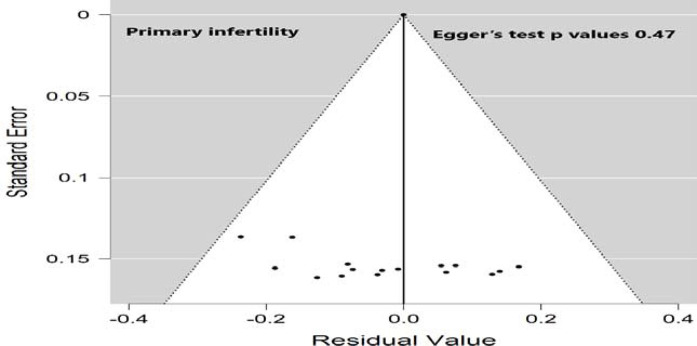
Funnel plot for assessing publication bias in the proportion of the primary infertility

**Figure 6.**
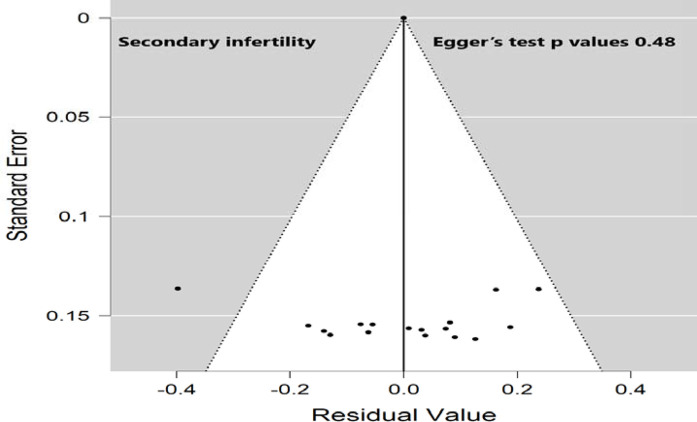
Funnel plot for assessing publication bias in the proportion of the secondary infertility

Given the very high heterogeneity level presented in the analyses of primary and secondary infertility, a subgroup analysis was performed to determine the effect of the publication year on the pooled prevalence of primary and secondary infertility (Table 2). The included studies were divided into three groups, and the results revealed that the studies published 2005 reported a relatively lower (44%, CI 15%–73%) pooled prevalence of primary infertility when compared with studies conducted between 2006 and 2015 and after 2016, which had pooled prevalence of 71% (CI 63%–79%) and 72% (CI 64%–79%), respectively. By contrast, the first group of studies showed a relatively higher pooled prevalence of secondary infertility (56%, CI 28%–85%) than the other two groups. In line with these results of the difference in the pooled prevalence among the three groups, the results revealed a difference in the heterogeneity among the three groups, where the first group (conducted before 2005) had a very high heterogeneity (I^2^ 99.6%) compared with the remaining groups. The publication bias for primary and secondary infertility analyses is presented in Figures 5 and 6, respectively, where the funnel plots for the two analyses were visually symmetric and Egger's test had *p* values of 0.47 and 0.48 for primary and secondary infertility analyses, respectively; therefore, these results did not provide evidence of a significant effect of publication bias.

### Pooled prevalence of infertility causes in Sudan

Table 3 shows the pooled prevalence for each cause of infertility in Sudan. In the total of 3822 cases presented in the included studies, female factors were responsible for 41% of infertility cases in Sudan, while male factors were responsible for 27% of infertility cases. The remaining causes were combined factors (male and female, 16%) and unexplained factors (17%).

### Causes of male and female infertility in Sudan

The pooled prevalence of causes of male and female infertility in Sudan using the random-effects model is presented in Table 4, and the results showed that azoospermia was the most common cause of male infertility in Sudan (37%). In addition, polycystic ovary syndrome (PCOS) was the most common cause of female infertility in Sudan with a pooled prevalence of 38%.

## Discussion

Ensuring healthy lives and promoting well-being for all ages is the third sustainable development goal,^32^ and the prevention and eradication of infertility fall under this goal. This goal cannot be achieved without proper understanding of the epidemiology of the disease.

This study clearly presents that the prevalence of infertility in Sudan is diminishing (2); the prevalence was 18% in the first infertility report in 1996^17^ and 10.4% in the most recent study in 2011.^18^ The decrease in the prevalence of infertility in Sudan over time is in line with Iranian findings.^33^ This result may be due to the relative progress in discovering new infertility diagnostic methods and treatments and introducing them in Sudan and the relative increase in the number of centers providing fertility services in Sudan.^10^

The present study reveals that the pooled prevalence of primary infertility was higher than that of secondary infertility in the Sudanese population (Figures 3 and 4). Some studies have found that the infertility type was associated with infections, especially sexually transmitted infections (STIs) and postpartum infections. If the prevalence of STIs and postpartum infections is high,^2,7,9–11^ the prevalence of secondary infertility is also increased. In Sudan, the prevalence of STI is lower than those in other regions of Sub-Saharan Africa,^34,35^ and this may explain the contrasting results of other infertility meta-analysis in Africa, where no significant difference was found in the prevalence of primary (49.9%) and secondary infertility (49.8%). Nevertheless, the same study found very similar results of primary and secondary infertility to the present study when a separate analysis was made for North Africa (70.6% and 29.6% for primary and secondary infertility respectively).^9,36^ The subgroup analysis of primary and secondary infertility by publication year reveals (Table 2) that the high heterogeneity level presented in this study is mainly attributed to studies that were published before 2005. Moreover, the prevalence of primary infertility was lower than that of secondary infertility in this group of studies compared with the remaining two groups. This could indicate that, over time, the occurrence of secondary infertility decreased due to the reduction of preventable infections and the availability of treatments, and this is in line with both Iranian and African findings.^9,33,36^

This study showed that infertility was mainly caused by female factors, followed by male factors, unexplained factors, and combined factors, including sexual dysfunction (Table 3). These results are in agreement with other meta-analysis findings reported by Eldib and Tashani;^36^ however, the pooled proportion of unexplained factors in this study (17%) was higher than those in other similar meta-analysis, i.e., 10.4% for Abebe et al. and 9% for Eldib and Tashani.^9,36^ This finding indicates the urgent need to take a deeper look at the unexplained factors in Sudan and to find some explanations for them.

A deeper look at the female-related factors showed that most infertility cases in Sudanese women caused by PCOS and other ovulatory conditions; however, this finding contradicts the results of Abebe et al., who found that pelvic inflammatory disease was the most common cause of female infertility. This difference may be caused by the difference in the prevalence of STIs among study populations.^2,7,9–11^ However, the exact cause of PCOS is still unknown,^37,38^ and this may be barrier to determining the cause of the high prevalence of PCOS in Sudanese women with infertility. This meta-analysis showed that most Sudanese men with infertility suffer from issues related to spermatic quality, especially azoospermia; by contrast, Abebe et al. found that oligospermia was the most common cause of male infertility.^9^ This result presents the urgent need to provide treatments to enhance the quantity and quality of sperm.

In most cases, infertility leads to various psychological problems that affect the individual and society; thus, this problem requires a serious, clear plan from all responsible individuals toward an effective intervention.^39^ Despite the significant increase in the number of fertility centers and the quality of its services in Sudan, it is still under the WHO-recommended standards^40^ and concentrated in only two states, namely, Khartoum and Gezira (Table1).^10^ In Sudan, the estimated total population according to 2020 data was more than 43 million with a population density of 25 per km^2^, and the ratio of medical staff per 1,000 people was 0.3, which is below the WHO standards that required at least 2.5 medical staff members for every 1,000 people.^41,42^ This high population density in addition to the very low medical staff to client ratio in Sudan indicates that the issue of infertility needs close attention from the healthcare policymakers and governments (federal and state government). To our knowledge, this is the first comprehensive quantitative meta-analysis summarizing available evidence to determine the pooled prevalence of infertility, its types, and etiology in Sudan.

### Strengths and limitations

This study used extensive and comprehensive search strategies and included published, unpublished, and gray literature. The study also used a standardized tool to evaluate the methodological quality of the studies. Studies with abstracts were the only ones included. The relatively small sample size of some studies is a limitation of this study and may raise questions about their representativeness. Moreover, no recent studies were included or available for this study.

## Conclusion

The results of this meta-analysis presented that the pooled prevalence of overall infertility, primary infertility, and secondary infertility in Sudan were 13%, 65%, and 35% respectively. Female factors were the most common causes of infertility in Sudan. The results also reveal a high prevalence of unexplained factors. The most common causes of female and male infertility in Sudan are PCOS and azoospermia, respectively. The interpretation of these findings should take into consideration the presence of substantial heterogeneity between the included studies.

### Registration and protocol

This review was developed based on the PRISMA guideline. The protocol of this review was registered by the International Prospective Register of Systematic Reviews (CRD42021253051).

### Support

None.

### Competing interests

The author(s) declared no potential competing of interest concerning the research, authorship, and/or publication of this article.

### Availability of data, code, and other materials

The data that support the review findings of this study are available upon submitting a reasonable request to the corresponding author.

### Acknowledgments

The authors would like to thank the Pan African University of Life and Earth sciences Institute, the African Union for the support, and the University of Ibadan for hosting this Ph.D. program.

## Figures and Tables

**Table 1:** Main characteristics of studies included in the meta-analysis

No	Authors and years	Location	Study design	Sample size	Infertility						

					IR (%)	PI (%)	SI (%)	FF (%) n	MF (%) n	CF (%) n	UF (%) n

1	Abdalla 2011^a^ [18]	Gezira	Cross-sectional	2000	10.4	77.4	22.6	(33.7)70	(25.5)53	(2.9)6	(38.0)79

2	Abdel Rahman 2003 [22]	Khartoum	Descriptive prospective study	124	10.4	57.2	42.8	(44.3)55	(30.6)38	0	(25)31

3	Elsheikh et al. 2002 [29]	National level	Cross-sectional	8469	11.5	79.5	20.5	(37)360	(20)195	NR	NR

4	Larsen. et al. 2000 [17]	National level	Cross-sectional	3093	18	16	84	NR	NR	NR	NR

5	Ericksen and Brunette 1996 [16]	National level	Cross-sectional	4791	12.3	23.5	76.5	NR	NR	NR	NR

6	Elussein et al. 2008 [12]	Khartoum	Cross-sectional	710	NR	62.4	37.6	(49.3)350	(36.2)257	(1.5)11	(13.0)92

7	Abdalla 2011^b^ [19]	Gezira	Cohort	200	NR	79.5	20.5	(37.5)76	(20.5)40	(31) 62	(11)22

8	Abdelrahman 2011 [23]	Khartoum	Cross-sectional	125	NR	NR	NR	(69.6)87	(20.8) 26	0	(9.6)12

9	Ahmed et al. 2015 [15]	Khartoum	Cross-sectional	191	NR	83.8	16.2	(25.1)48	(42.9)82	(3.7)7	(28.3)54

10	Geaballah 2017 [27]	Khartoum	Cross-sectional	110	NR	82.7	17.3	(32.7)36	(20.9)23	(33.6)37	(12.7)14

11	Abbas 2017 [25]	Khartoum	Cross-sectional	300	NR	NR	NR	(33.9)102	(34.7)104	(6.7)20	(24.7)74

12	Elhussein et al. 2019 [11]	Khartoum	Cross-sectional	800	NR	68.9	31.1	(42.8)342	(35.5)284	(18.4)147	(3.4)27

13	Ahmed et al. 2009 [21]	Gezira	Cross-sectional	194**	NR	53.2	46.8	NR	NR	NR	NR

14	Nabag et al. 2011 [28]	Khartoum	Cross-sectional	205*	NR	76	24	NR	NR	NR	NR

15	Yasin and Elnimeiri 2013 [4]	Khartoum	Cross-sectional	162*	NR	66	34	NR	NR	NR	NR

16	Husain 2017 [26]	Gezira	Cross-sectional	97*	NR	88.7	11.3	NR	NR	NR	NR

17	Albalola 2018 [24]	Kordofan	Cross-sectional	125*	NR	68.8	31.2	NR	NR	NR	NR

18	Abdallah 2019 [20]	Gezira	Cross-sectional	125*	NR	68.8	31.2	NR	NR	NR	NR

19	Hussein et al. 2019 [15]	Khartoum	Cross-sectional	143*	NR	60.8	39.2	NR	NR	NR	NR

20	Omer et al. 2020 [13]	Gezira	Cross-sectional	415*	NR	63.8	36.2	NR	NR	NR	NR


Abbreviations: *Female; **Male; *CF*, combined factor; *FM*, female factor; *MF*, male factor; *N*, number; *NR*, not reported; *PI*, primary infertility; *SI*, secondary infertility; *UF*, unexplained factor.

**Table 2:** Subgroup analysis of primary and secondary infertility by publication year

		Number of studies	Pooled proportion (95% CI)	Heterogeneity (I^2^ %, *p* value)

Year of publication (PI)	Before 2005	4	44(15–73)	99.6, *p* < 0.001

	2006–2015	7	71(63–79)	93.4, *p* < 0.001

	2016–2021	7	72(64–79)	91.2, *p* < 0.001

Year of publication (SI)	Before 2005	4	56(27–85)	99.6, *p* < 0.001

	2006–2015	7	29(21–37)	93.4, *p* < 0.001

	2016–2021	7	28(21–36)	91.2, *p* < 0.001


*PI*, primary infertility; *SI*, secondary infertility; *CI*, confidence interval

**Table 3:** Summary table of the data from included studies showing the pooled prevalence of the causes of infertility using the random-effects model

Causes of infertility	Number of studies	Pooled sample size	Pooled prevalence % (95% CI)	Heterogeneity (I^2^ %, *p* value)

Female factors	11	3822	41(34-48)	94.4, *p* < 0.001

Male factors	11	3822	27(22-33)	92.6, *p* < 0.001

Combined factors	8	2599	16(6-26)	99.1, *p* < 0.001

Unexplained factors	10	2848	17(10-24)	96.8, *p* < 0.001


*CI*, confidence interval

**Table 4:** Summary table of the data from included studies showing the pooled prevalence of the causes of male and female infertility using the random-effects model

Causes of male infertility	Number of studies/Pooled sample size	Pooled prevalence % (95% CI)	Causes of female infertility	Number of studies/Pooled sample size	Pooled prevalence % (95% CI)

Azoospermia	7/1056	37(24–50)	Ovulatory factor	5/1193	36(25–47)

Oligozoospermia	7/1056	30(16–43)	Tubal factor	9/1826	22(13–31)

Asthenozoospermia	4/689	30(17–43)	Uterine factor	8/1675	15(6–24)

Mixed pathology/others	4/746	27(10–44)	PCOS	8/1738	38(21–55)

			Mixed pathology/others	9/1573	21(9–33)


*CI*, confidence interval; *PCOS*, polycystic ovary syndrome
